# Application of Bioengineered Materials in the Surgical Management of Heart Failure

**DOI:** 10.3389/fcvm.2019.00123

**Published:** 2019-08-20

**Authors:** Simranjit S. Pattar, Ali Fatehi Hassanabad, Paul W. M. Fedak

**Affiliations:** Section of Cardiac Surgery, Department of Cardiac Sciences, Cumming School of Medicine, Libin Cardiovascular Institute of Alberta, University of Calgary, Calgary, AB, Canada

**Keywords:** extracellular matrix, biomaterials, epicardium, heart failure, cardiac surgery, bioscaffold, myocardial infarction

## Abstract

The epicardial surface of the heart is readily accessible during cardiac surgery and presents an opportunity for therapeutic intervention for cardiac repair and regeneration. As an important anatomic niche for endogenous mechanisms of repair, targeting the epicardium using decellularized extracellular matrix (ECM) bioscaffold therapy may provide the necessary environmental cues to promote functional recovery. Following ischemic injury to the heart caused by myocardial infarction (MI), epicardium derived progenitor cells (EPDCs) become activated and migrate to the site of injury. EPDC differentiation has been shown to contribute to endothelial cell, cardiac fibroblast, cardiomyocyte, and vascular smooth muscle cell populations. Post-MI, it is largely the activation of cardiac fibroblasts and the resultant dysregulation of ECM turnover which leads to maladaptive structural cardiac remodeling and loss of cardiac function. Decellularized ECM bioscaffolds not only provide structural support, but have also been shown to act as a bioactive reservoir for growth factors, cytokines, and matricellular proteins capable of attenuating maladaptive cardiac remodeling. Targeting the epicardium post-MI using decellularized ECM bioscaffold therapy may provide the necessary bioinductive cues to promote differentiation toward a pro-regenerative phenotype and attenuate cardiac fibroblast activation. There is an opportunity to leverage the clinical benefits of this innovative technology with an aim to improve the prognosis of patients suffering from progressive heart failure. An enhanced understanding of the utility of decellularized ECM bioscaffolds in epicardial repair will facilitate their growth and transition into clinical practice. This review will provide a summary of decellularized ECM bioscaffolds being developed for epicardial infarct repair in coronary artery bypass graft (CABG) surgery.

## Introduction to Heart Failure

Heart failure is a chronic and progressive condition characterized by maladaptive structural cardiac remodeling and poor cardiac pump function. The most common cause of heart failure is damage to the cardiac muscle caused by ischemic injury, otherwise known as myocardial infarction (MI) (1). There are an increasing number of individuals living with heart failure, with 960,000 new cases reported each year in the US alone (2). As a result, an estimated >8 million individuals will be living with heart failure in the US by 2030 (3). Surgical revascularization remains the primary treatment modality for patients who have suffered from an MI. However, nearly one in every five heart failure patients is readmitted for heart failure or other related causes within 30 days (1). Therefore, heart failure is often referred to as a “revolving door condition” due to high rates of readmission. Given its increasing prevalence and high rate of readmission, it is necessary to improve our understanding of the surgical management of heart failure. Extracellular matrix (ECM) bioscaffolds may be the key to unlocking the potential of the epicardial surface of the heart with the ultimate goal of driving endogenous mechanisms of repair and attenuating progressive heart failure following ischemic injury.

## The Current Surgical Management of Heart Failure

According to current guidelines, the primary objective in the surgical management of MI is to restore blood flow to the infarct region in order to preserve myocardial viability and alleviate symptoms (4, 5). Revascularization may be achieved by coronary artery bypass graft (CABG) surgery or percutaneous coronary intervention (PCI), and has been shown to improve survival in patients (4–8). A recent meta-analysis including twenty-one studies and 16,191 patients found revascularization by CABG or PCI was beneficial compared to medical treatment alone in patients suffering from ischemic heart disease and reduced left ventricular ejection fraction (LVEF) (9). The survival benefits of surgical revascularization are clear, and the completeness of revascularization is integral to preserving myocardial viability (4). Despite surgical intervention, studies have documented 12 and 22% readmission rates in patients who have undergone CABG or PCI, respectively, due to heart failure (6, 10).

For patients who progress to end-stage clinical heart failure, cardiac transplantation remains the gold standard treatment modality (11, 12). However, limited donor supply and an increasing number of eligible patients for cardiac transplants have driven the need for innovative alternative strategies. Mechanical circulatory support (MCS), specifically the left ventricular assist device (LVAD), has vastly improved since the seminal work of Dr. Michael E. DeBakey, Dr. Denton A. Cooley, and others (13–16). MCS may be utilized as a bridge to recovery, bridge to transplantation, or as a destination therapy in patients who are ineligible for cardiac transplantation (17, 18). INTERMACS (Interagency Registry for Mechanically Assisted Circulatory Support) includes >15,000 patients from 158 hospitals; it reports a 1-year survival rate of 80% and 2-year survival rate of 70% in patients receiving a continuous-flow device (17, 19). Importantly, despite the improvements made in MSC technology, a number of complications are still associated with its use (19–22). Adverse events reported at 2-year follow up of 133 patients treated using a continuous flow LVAD include, bleeding requiring blood transfusion (81%), cardiac arrhythmia (56%), right-sided heart failure (20%), LVAD-related infection (35%), stroke (18%), LVAD-thrombosis (4%), and pump replacement (9%) (23). Therefore, an opportunity exists to better understand and address the underlying cellular and molecular causes of heart failure in order to improve the prognosis of patients with heart failure.

## Taking a Stem Cell-Based Approach

An array of stem cell-based approaches have emerged in the past two decades with the aim of restoring myocardial function and preventing the progression of heart failure. Despite the numerous clinical trials conducted and their important contribution to our current understanding of the treatment of heart failure following MI, an efficacious stem cell-based therapy with clinically relevant outcomes has yet to emerge (24–26). Clinical trial data regarding the use of a stem cell-based approach in patients suffering from acute myocardial infarction or ischemic cardiomyopathy remains variable and inconclusive (24, 25, 27).

In the case of acute myocardial infarction, earlier trials such as BOOST (BOne marrOw transfer to enhance ST-elevation infarct regeneration) and REPAIR-AMI (Reinfusion of Enriched Progenitor Cells and Infarct Remodeling in Acute Myocardial Infarction) showed improved LVEF in patients treated with bone marrow mononuclear cells (BM-MNCs) (28–30). However, these improvements were not replicated in future studies using BM-MNCs, including but not limited to, BOOST-2 (BOne marrOw transfer to enhance ST-elevation infarct regeneration-2), LATE-TIME (Late Timing in Myocardial Infarction Evaluation), and SWISS-AMI (SWiss multicenter Intracoronary Stem cells Study in Acute Myocardial Infarction) (24, 31–33).

Similarly, in the case of ischemic cardiomyopathy, patients with advanced heart failure who received BM-MNC therapy displayed a 9% improvement in LVEF at 4-months follow-up compared to baseline (34). However, the FOCUS-CCTRN (First Mononuclear Cells injected in the United States conducted by the CCTRN) trial showed a modest 2.7% LVEF improvement in patients treated with BM-MNC compared to placebo, alongside no significant improvement in infarct size (35). Furthermore, CHART-1 (Congestive Heart Failure Cardiopoietic Regenerative Therapy) found no significant difference amongst advanced heart failure patients (*n* = 157) receiving cardiopoietic cell therapy compared to sham control (*n* = 158) at 39-weeks follow-up (24, 36).

In addition to the variable clinical trial results discussed, poor stem cell engraftment at the site of delivery and the inherent conflict of introducing stem cells to an injured and hostile cardiac environment present significant hurdles to directing optimal cell function and tissue recovery (37, 38). The recent controversy surrounding c-kit^+^ cardiac stem cells and interruption of the CONCERT-HF (Combination of Mesenchymal and C-kit+ Cardiac Stem Cells as Regenerative Therapy for Heart Failure) trial has significantly contributed to uncertainty regarding the clinical efficacy of this approach (39–45).

## The Paracrine Hypothesis

Despite the challenges associated with a stem cell-based approach, it has become clear that stem cells largely exert their effects in a paracrine fashion (41, 46–53). The ability of bone marrow-derived cells to produce a potent angiogenic growth factor response, enhance endothelial cell proliferation, and improve perfusion and function in models of ischemic injury has been described (49, 53–55). Specifically, autologous bone marrow cells are capable of producing a vascular endothelial growth factor (VEGF)-dependent response that drives angiogenesis and improves perfusion in a porcine model of MI (54). Moreover, human mesenchymal stem cell conditioned media (MSC-CM) has been shown to reduce MI size, enhance capillary density, and improve overall cardiac function compared to control media using a porcine model of MI (56, 57). Overall, it is the paracrine factors produced that play a fundamental role in mediating the effects of stem cell therapy.

**Figure 1:**
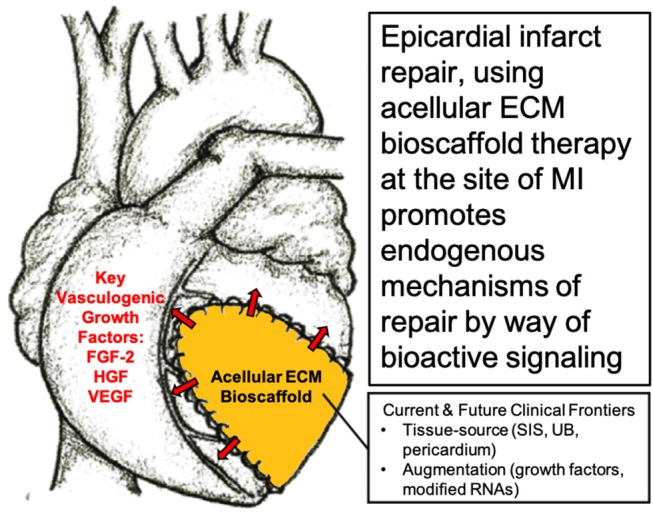
Surgical implantation of acellular extracellular matrix (ECM) bioscaffold on the epicardial surface of the heart promotes endogenous mechanisms of repair by way of bioactive paracrine signaling. Image, in part, provided courtesy of Dr. Holly Mewhort, Section of Cardiac Surgery, Department of Cardiac Sciences, Cumming School of Medicine, Libin Cardiovascular Institute of Alberta, University of Calgary, Calgary, Alberta, Canada.

The question, therefore, becomes “*is the delivery of cells necessary for a therapeutic effect?”* Given the challenges associated with a stem cell-based approach, our research group alongside many others have directed their attention toward modulating the local cardiac microenvironment and paracrine response, without the administration of stem cells, in order to direct endogenous mechanisms of cardiac repair (48, 51, 57–62). In particular, our research group has shown the benefits of using acellular bioactive extracellular matrix (ECM) scaffolds for epicardial infarct repair (63–66). By providing an optimal ECM microenvironment with the necessary paracrine growth factors, the aim is to limit infarct expansion by attenuating cardiac fibrosis and to promote vasculogenesis in order to improve blood flow to the infarcted myocardium (63, 65, 67) (Figure 1).

## Targeting the Epicardium in Cardiac Surgery

A unique opportunity exists to enhance endogenous repair mechanisms of the heart at its epicardial surface. In the case of routine cardiac surgery, surgeons gain access to the heart by way of sternotomy followed by cardiotomy, at which point they are presented with the epicardial surface of the heart. The epicardium is a promising anatomic niche that is involved in early cardiac development, the production and regulation of ECM components, paracrine signaling, and response to ischemic injury (68).

**Figure 2:**
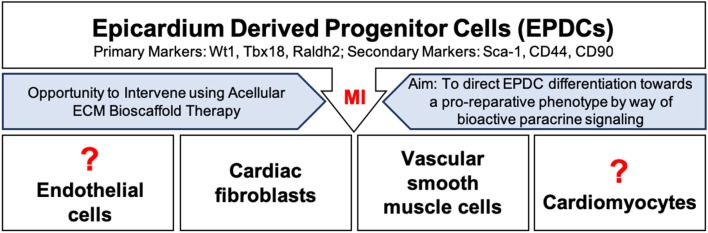
Epicardium derived progenitor cell (EPDC) activation, mobilization, and differentiation following myocardial infarction. EPDCs may differentiate into cardiac fibroblasts, vascular smooth muscle cells, or possibly into endothelial cells or cardiomyocytes. Intervention using acellular ECM bioscaffold therapy may be used to direct EDPCs toward a pro-reparative, vasculogenic fate.

This thin outermost mesothelial layer of the heart contributes to normal cardiac development as it gives rise to multipotent cardiac progenitor cells, called epicardium derived progenitor cells (EPDCs) (68, 69). EPDCs have been found to differentiate to coronary vascular smooth muscle cells and cardiac fibroblasts by way of epithelial to mesenchymal transition (EMT) (70–73) (Figure 2). Studies have also reported EPDC differentiation to endothelial cell and cardiomyocyte populations (74–77). However, the contribution of EPDCs to endothelial cell and cardiomyocyte populations remains highly debatable and further lineage tracing is warranted to ascertain the extent of this contribution (71, 73, 77–82) (Figure 2).

During early cardiac development, the epicardium dictates myocardial maturation and compaction, and the formation of the coronary vasculature and Purkinje fibers (68, 83–85). Beyond its contribution to various cell populations, the epicardium has been shown to be a key player involved in paracrine signaling and the modulation of ECM components (82, 86–90). This makes the epicardium an ideal candidate that may be targeted to manipulate ECM remodeling and promote endogenous repair and regeneration of the adult human heart.

## Ischemic Injury Leads to ECM Remodeling

Normally, the epicardium is quiescent in the healthy adult human heart, yet it may hold great regenerative capacity (68, 83, 91, 92). Following an MI, EPDCs become activated and migrate to the site of injury, where they have been shown to largely differentiate into vascular smooth muscle cells or fibroblasts (70, 92, 93) (Figure 2). The expression of markers including, Wilms tumor protein (Wt1), T-box transcription factor 18 (Tbx18), and retinaldehyde dehydrogenase 2 (Raldh2), has been reported in activated EPDC populations (69, 82, 92). Recent findings have further highlighted the heterogeneity within EPDC populations following an MI based on the differential expression of stem-cell antigen 1 (Sca-1), CD44, and CD90; these subpopulations may present clinically relevant targets (94). Overall, activation of the epicardium has been shown to play an important role in supporting the development of new vasculature by way of a robust paracrine response (79, 92). EPDC derived conditioned media has been reported to induce functional recovery in a mouse model of MI by way of a robust fibroblast growth factor-2 (FGF-2) and VEGF mediated angiogenic response (92). Clearly, an opportunity exists to direct EPDCs toward a pro-reparative or pro-vasculogenic fate, and away from a pro-fibrotic fate. As mentioned above, the completeness of revascularization is critical to the preservation of myocardial viability following an MI (4). Therefore, the aim is to promote vasculogenesis in order to improve blood flow to the infarcted myocardium, and to limit infarct scar expansion by attenuating the activity of cardiac fibroblasts.

Cardiac fibroblasts play an essential role in normal heart function, not only structurally and mechanically, but with regards to the biochemical and electrical properties of the cardiac environment as well (95–99). In the event of an ischemic injury and the resulting disruption of the local microenvironment of the infarcted myocardium, these fibroblasts become activated. Activated fibroblasts, known as myofibroblasts, are the key mediators of ECM remodeling (96, 98, 100, 101). Fibrotic remodeling of the infarcted myocardium is exacerbated by the migration and activation of additional cardiac fibroblasts at the site of injury. Of note, the epicardium is a significant source of these migratory cardiac fibroblasts (79, 82, 92, 93, 102–104). Myofibroblast activity at the site of MI leads to dysregulation of ECM homoeostasis, resulting in the deposition of a collagenous scar (98, 104, 105). Initially, this response is crucial in preventing ventricular free wall rupture at the site of an MI. However, persistent myofibroblast activation when left unchecked leads to infarct scar expansion, diastolic, and systolic dysfunction due to structural cardiac remodeling, and eventually end-stage clinical heart failure (97, 98, 105–108).

Therefore, targeting the epicardium post-MI by providing the necessary bioinductive cues capable of promoting a pro-reparative phenotype rather than a pro-fibrotic phenotype is compelling. Our research group and others believes that acellular ECM bioscaffolds offer an ideal approach by which the post-MI cardiac environment may be directed toward a pro-reparative phenotype by way of bioactive signaling (Figures 1, 2).

## Bio-Engineered Materials for Epicardial Infarct Repair

Collagen is commonly studied for ECM bioscaffold-based infarct repair as it is the primary component of the cardiac ECM (109–111). Acellular type I collagen cardiac bioscaffold has been shown to preserve contractility, reduce cardiac fibrosis, and attenuate LV remodeling using a murine model of MI (112, 113). Additionally, acellular type I collagen cardiac bioscaffold therapy has been reported to promote a pro-vasculogenic response, which is accompanied by increased vessel density in the injured heart (112–115) Other natural bioscaffolds, such as fibrin, gelatin, Matrigel, alginate, and chitosan-based scaffolds, have also been investigated for infarct repair and regeneration (111, 116–119). Acellular fibrin-based scaffolds leverage the blood clotting cascade to polymerize *in situ*, and have been reported to preserve cardiac function and improve neovascularization in a rat model of MI (111, 120, 121). Despite the potential of these acellular strategies, there are challenges associated with the use of natural bioscaffolds, such as rapid degradation and poor mechanical performance (111, 122).

Promising results have also been found using synthetic scaffold solutions, which typically include the use of polylactic acid (PLA), polyglycolic acid (PGA), poly-ε-caprolactone (PCL), polyester urethane urea (PEUU), polytetrafluoroethylene (PTFE), or varying combinations of the aforementioned materials (110, 122, 123). For example, polyester urethane urea (PEUU) scaffold implantation in a rat model of MI is capable of improving overall contractile function and cardiac remodeling (124). While the mechanical properties of synthetic scaffold solutions are highly tunable, they lack the biological complexity required to target the epicardial surface of the heart by way of paracrine signaling and bioactive factors.

As such, the development of decellularized tissue-derived ECM bioscaffolds remains a focal point within the field. These acellular ECM bioscaffolds facilitate directing endogenous mechanisms of repair and regeneration at the site of ischemic injury by way of bioactive paracrine signaling (63–65, 109, 125–128) (Figure 1). Acellular ECM bioscaffolds retain the native ECM architecture and composition, including a variety of embedded growth factors, of the tissue from which they were derived (125, 126, 129). These complex bioscaffolds may be exploited to provide an optimal microenvironment capable of enhancing blood flow and attenuating cardiac myofibroblast activity at the site of an MI. Of the tissue-derived ECM bioscaffolds, acellular porcine-derived small intestinal submucosa (SIS) ECM is best characterized in the literature with regards to epicardial infarct repair. We have previously described the collection of acellular ECM bioscaffolds available for a variety of indications in cardiac surgery (98).

The small intestine is a highly vascularized organ and therefore the composition and structure of SIS-derived ECM bioscaffold is proposed to be highly conducive to revascularization itself (109, 126). Specifically, 90% of SIS-ECM bioscaffold is type I collagen, fibronectin, laminin, and glycosaminoglycans (GAGs) ECM components (109, 130). The role of fibronectin and laminin in endothelial cell adhesion and the maintenance of vascular structures, respectively, has been characterized (109, 131–133). Additionally, GAGs play an important role in binding the growth factors and cytokines within the ECM and therefore present a possible target for modifying tissue-derived ECM bioscaffolds with signaling factors (109, 134). Finally, SIS-ECM itself has been shown to naturally contain essential growth factors, both bound by GAGs and embedded within the ECM itself, including fibroblast growth factor-2 (FGF-2), VEGF, and hepatocyte growth factor (HGF), which play key roles in vasculogenesis (109, 135).

**Figure 3:**
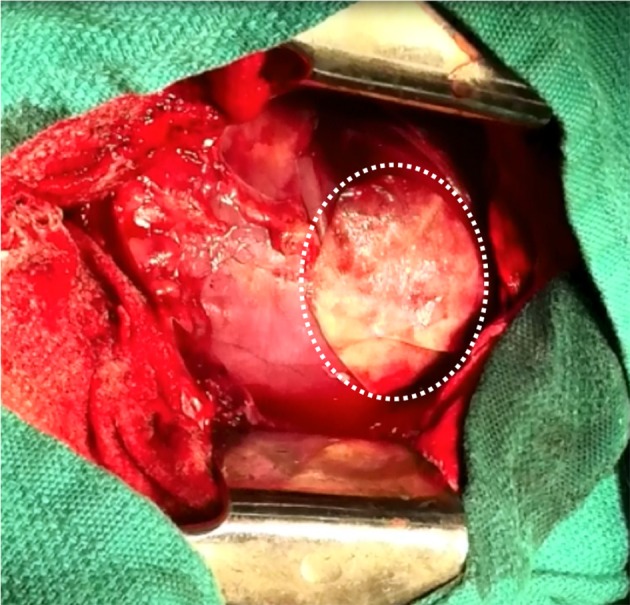
Surgical implantation of porcine-derived acellular small intestine submucosa (SIS) extracellular matrix (ECM) bioscaffold (CorMatrix® ECM, CorMatrix Cardiovascular Inc., USA) over the site of ischemia-reperfusion injury on the anterior wall of the left ventricle, in a porcine model. Image provided courtesy of the Fedak Research Group (Dr. Holly Mewhort and Jeannine Turnbull) Campbell Family Cardiac Translational Laboratory, Libin Cardiovascular Institute of Alberta, University of Calgary, Calgary, Alberta, Canada.

In the context of epicardial infarct repair, our research group has highlighted the promise of acellular SIS-ECM bioscaffold, CorMatrix® ECM (CorMatrix Cardiovascular Inc., USA) (63–66). We have shown that the interaction of human cardiac fibroblasts with CorMatrix® ECM results in a robust fibroblast growth factor-2 (FGF-2) dependent cell-mediated paracrine response capable of stimulating new blood vessel assembly (65). This is recapitulated *in vivo*, using a rat MI model: animals treated via surgical implantation of CorMatrix® ECM post-MI compared to animals treated with sham or inactivated CorMatrix® ECM displayed an FGF-2-dependent increase in vascularity, reduced LV dilatation, improved ejection fraction, and improved contractility (65). Further studies using a large pre-clinical porcine ischemia-reperfusion model have similarly demonstrated that surgical implantation of CorMatrix® ECM improves vascularity and functional recovery of the infarct region (63, 64) (Figure 3). Other groups have assessed SIS-ECM bioscaffold in surgical reconstruction of septal defects, vascular or outflow tract augmentation, and valve reconstruction, and have yielded positive results (136). However, long-term patient follow up is required to truly understand the impact of this intervention. Notably, our research group is characterizing the clinical use of commercially-available CorMatrix® ECM in an on-going first-in-human phase I clinical trial (NCT02887768) for epicardial infarct repair at the time of surgical revascularization (CABG surgery).

Beyond SIS-ECM bioscaffold therapy for epicardial infarct repair, acellular ECM bioscaffolds derived from other tissue sources, such as the urinary bladder (UB), amniotic membrane (AM), and cardiac tissue have been investigated (137–140). Given the fact that ECM bioscaffolds are a function of the physiological requirements of the tissue from which they are derived, the tissue source will influence each bioscaffold's ability to direct cardiac repair. UB-ECM bioscaffold has been reported to outperform PTFE synthetic scaffold, and displays favorable tissue integration and replacement in a porcine MI model (139). More recently, an acellular pericardium-derived ECM bioscaffold was shown to support neovascularization and neoinnervation, alongside improved LVEF, cardiac output, and reduced infarct size in a porcine MI model at 30-day follow-up (137). Future work should continue to investigate acellular ECM bioscaffolds derived from various tissue sources in the context of epicardial infarct repair.

Additionally, augmentation of the aforementioned bioscaffolds with additional growth factors and/or modified RNAs, may also play an important role in modulating the local paracrine environment and enhancing their therapeutic effect. While the specific details and challenges are beyond the scope of this review, modified RNAs may be utilized to promote angiogenesis following an MI (141–143). Intramyocardial injection of VEGF-A modified RNA has been reported to enhance Wt1^+^-EPDC to endothelial cell differentiation and to promote functional vessel formation in a mouse model of MI (143). Similarly, our research group has shown that enhancement of SIS-ECM with additional FGF-2 further improves ECM homeostasis and cardiac function in a rat model of MI (63, 66). Overall, acellular tissue-derived ECM bioscaffolds are a promising therapeutic strategy for the modulation of cardiac repair at the site of an MI. The important role that bioactive paracrine signaling plays in directing endogenous mechanisms of repair at the site of an MI should remain a focal point moving forward.

## Conclusion

A unique opportunity exists to augment surgical revascularization via CABG surgery using acellular ECM bioscaffold therapy. The epicardial surface is readily accessible in open heart surgery. As it is an anatomic niche responsible for normal cardiac development and paracrine signaling, which becomes activated in response to ischemic injury, we can leverage the epicardium to direct endogenous repair at the site of an MI. Acellular ECM bioscaffold therapy has been shown to improve vascularization and attenuate cardiac fibroblast-mediated ventricular remodeling following an MI by way of its bioactive signaling. By providing an optimal ECM bioscaffold signaling environment the goal is to shift the damaged cardiac tissue toward a pro-reparative phenotype, and away from a pro-fibrotic phenotype, and to improve overall revascularization of the tissue. Overall, by targeting the underlying cellular and molecular causes of heart failure using acellular ECM bioscaffold therapy, this innovative strategy may be able to significantly improve the prognosis of patients who have suffered from an MI.

## Author Contributions

SP, AF, and PF designed, drafted, and revised the manuscript.

### Conflict of Interest Statement

The authors declare that the research was conducted in the absence of any commercial or financial relationships that could be construed as a potential conflict of interest.
